# Hypoxanthine enhances the cured meat taste

**DOI:** 10.1111/asj.12625

**Published:** 2016-05-12

**Authors:** Sayaka Ichimura, Yukinobu Nakamura, Yuka Yoshida, Akihito Hattori

**Affiliations:** ^1^ Japan Meat Science and Technology InstituteTokyoJapan

**Keywords:** *cured meat taste*, *hypoxanthine*, *nitrite*, *sarcoplasmic fraction*, *sensory analysis*

## Abstract

We evaluated the enhancement of cured meat taste during maturation by sensory analysis. We focused on the heat‐stable sarcoplasmic fraction (HSSF) to identify the factors related to cured meat taste. Because the dry matter of HSSF contained more than 30% nitrogen, nitrogen compounds such as free amino acids, small peptides and adenosine triphosphate‐related compounds seemed to be the important components of HSSF. The samples cured with HSSF for 2 h exhibited the same taste profile as ones cured without HSSF for 168 h. Therefore, the changes in the amount and fractions of nitrogen compounds were examined in HSSF during incubation from 0 to 168 h. The concentration of hypoxanthine (Hx) gradually increased, while inosine‐5′‐monophosphate decreased during the incubation. The samples cured with pickles containing various concentrations of Hx were subjected to sensory analysis. The addition of Hx, in a dose‐dependent fashion, enhanced cured meat taste by maturation for 2 h. It was concluded that Hx is essential for the enhancement of cured meat taste.

## Introduction

Curing is an essential process in the manufacture of meat products such as hams and sausages. In the curing process, the curing ingredients, usually salt, polyphosphate and nitrite, are added to meat. There are two basic curing techniques, called dry curing and brine curing. In dry curing, the mixture of curing ingredients is directly rubbed over the surface of the meat. On the other hand, in brine curing, meat is immersed in the curing solution or pickle. In the manufacture of hams in Japan, brine curing is commonly employed.

It has been found that curing contributes to water‐holding capacity, binding, the fixation of the characteristic cured meat color, the inhibition of the growth of certain bacteria and the development of a specific cured meat flavor. Nitrite is essential for fixation of the characteristic meat color (Fox 1987), the inhibition of the growth of bacteria (Perigo *et al*. 1967) and the development of a specific cured meat flavor (Cho & Bratzler 1970; Brown *et al*. 1974; Macdonald *et al*. 1980).

Cured meat flavor common to cured meat products is distinguishable from that of raw or cooked meat and gradually increases during maturation. The hams manufactured in Europe such as Parma ham and Jamon serrano have a strong cured meat flavor induced by the dry curing process over 1 year. The flavor of cured meat products manufactured in Japan also has the same tendency to increase during the maturing periods. The Ministry of Agriculture, Forestry and Fisheries of Japan has defined ‘matured meat products’ which are required for maturing periods over a certain level, as the specific standards in the Japan Agricultural Standards (JAS).

In general, the flavor of food is composed of aroma and taste. With respect to the aroma of the cured meat flavor, some specific volatile compounds have been identified (e.g. 4‐methyl‐1, 2‐pentanone, 2, 2, 4‐trimethyl‐hexane, 1, 2, 4‐trimethyl‐cyclohexaneand 1, 3‐dimethylbenzene) (Ramarathnam *et al*. 1991a, 1991b). It was also found that nitrite inhibited the development of off‐flavors resulting from lipid oxidation in heated meat (Sato & Hegarty 1971; Igene *et al*. 1985).

On the other hand, there are no reports focused, not on flavor, but taste of cured meat, whereas many taste components, which are nitrogen compounds such as free amino acids, small peptides and adenosine triphosphate (ATP)‐related compounds, lactic acid, sugar and minerals, exist in raw meat. Interestingly, there are a few reports on components and mechanisms involved in the improvement of meat taste during post mortem aging (Nishimura *et al*. 1988; Nishimura 1998). The improvement of meat taste during post mortem aging is related to the increase of free amino acids and small peptides in raw meat.

Thus, there is a possibility that the sarcoplasmic fraction containing nitrogen compounds, such as amino acids‐related and ATP‐related compounds, contributes to the enhancement of cured meat taste. Therefore, we focused on the sarcoplasmic fraction to identify the factors related to taste specific for cured meat in this study. A better understanding of the mechanisms of taste development will likely lead to a reduction of the maturing period necessary for the development of enhanced cured meat taste and/or the improvement of the desirable qualities of cured meat products.

The purpose of this study was to clarify and identify the factors involved in the enhancement of cured meat taste during maturation by evaluation using sensory analysis. Sensory analysis by highly skilled and well‐trained panelists may be highly effective in the evaluation of the taste of cured meat. In previous studies, cured meat flavor was evaluated by sensory analysis, with flavor being described by a combination of aroma and taste (Macdonald *et al*. 1980; Shahidi *et al*. 1986). Aroma and taste of food are sensed by ‘olfaction’ stimulated by volatile compounds, and ‘gustation’ stimulated by water‐soluble compounds, such as free amino acids and ATP‐related compounds, respectively. To distinguish the aroma and the taste exactly, both sensory analyses must be separately evaluated.

Thus, the following experiments were performed with sensory analysis excluding the influence of the aroma: (i) examination of the effects of the sarcoplasmic fraction on enhanced cured meat taste prepared in a model brine curing system; and (ii) identification of the compounds related to the cured meat taste products by focusing on the changes of nitrogen compounds in the heat‐stable sarcoplasmic fraction (HSSF).

## Materials and Methods

### Materials

Chilled loins from one side of six Berkshire pig carcasses were obtained from a local meat retailer. The Longissimus thoracis muscle was separated from loins. Two of six separated muscles were sliced to 5 mm thickness for sensory analysis and the others were minced for preparation of the sarcoplasmic fraction and chemical analysis.

### Chemicals

Sodium nitrite, inosine monophosphate (IMP), inosine (INO) and hypoxanthine (Hx) standards were purchased from Wako Pure Chemical Industries, Ltd. (Osaka, Japan). These chemicals were used in Wako Special Grade.

### Preparation of the HSSF

Minced meat was mixed with an equal weight of 0.1 mol/L NaCl solution. The mixture was moderately stirred by a glass rod and centrifuged at 3000 × *g,* 4°C for 10 min. The supernatant, sarcoplasmic fraction, was heated at 100°C for 20 min and then centrifuged at 3000 × *g,* 4°C for 10 min. The resultant supernatant was used as the HSSF in the sensory analysis and chemical analysis.

### Preparation of samples for sensory analysis

The sliced meats were cured with a pickle consisting of 2% NaCl and 200 ppm NaNO_2_ at 4°C. To determine the effects of maturing periods, HSSF or Hx, the samples were treated as follows: the effect of maturing periods was examined by the samples cured for 2, 72 or 168 h; the effect of HSSF was examined by the samples cured with the pickle containing 50% (v/v) of HSSF for 2 h; the effect of Hx was examined by the samples cured with the pickle containing Hx at concentrations of 0, 4, 8 and 16 µmol/mL pickle for 2 h. The concentration of NaCl in the pickle containing HSSF was also adjusted to 2%. In all experiments, the weight ratio of meat samples to pickle was 1:1, the samples were packed into an S‐WRAP bag (Star Plastic Industry Inc., Osaka, Japan), heated in a water bath at 70°C for 20 min and then immediately immersed in ice water. The cured samples were brought to room temperature then subjected to sensory analysis.

### Sensory analysis

Only the cured meat taste was evaluated by closing the nostrils of panelists using commercial nose clips, with the aim of excluding the influence of the aroma. The cured meat taste was defined as the specific taste present only on meat with curing, but not present on meat without curing. To acquire the ability to identify the specific taste, the panelists were repeatedly trained in the sensory analysis of meat with or without curing at the Japan Meat Science and Technology Institute, Tokyo, Japan. Meat cured with the pickle consisting of 2% NaCl and 200 ppm NaNO_2_ at 4°C for 2 h was used as the standard sample. The strength of the taste of each sample prepared for the sensory analysis was compared with that of the standard samples. The strength of the taste was evaluated by scoring in five steps. The scores of ‐2, ‐1, 0, +1 and +2 correspond to very weak, weak, equal, strong and very strong compared to the standard, respectively.

Sensory analysis was performed and repeated two times in each analysis by the well‐trained panelists composed of four men and two women (age 25 to 48 years) at the Japan Meat Science and Technology Institute.

### Chemical analysis

Minced meat was mixed with an equal weight of 2% NaCl solution, and then incubated at 10°C for 0, 72 and 168 h. The temperature was the upper limit established in the specific JAS Standards.

A part of the incubated meat was used for determination of nitrogen compounds. Distilled water was added to the incubated meat to adjust for the concentration of NaCl to 0.1 mol/L and then the mixture was homogenized using a homogenizer (Biomizer BM‐1; Nissei Co., Ltd. Tokyo, Japan) and centrifuged. The nitrogen concentration of the supernatant was determined by Dumas combustion method (SUMIGRAPH NC‐220 F; Sumika Chemical Analysis Service Co., Ltd, Tokyo, Japan). Elemental nitrogen after complete combustion of organic substances in the supernatant was detected by TCDthermal conductivity detector gas chromatography. The fractionation of nitrogen compounds in the supernatant was carried out by gel filtration chromatography. The supernatant was filtered using a 0.45 µm cellulose membrane. The filtrate was applied to a high‐performance liquid chromatography (HPLC) system (Waters2695; Waters Co., Ltd, Tokyo, Japan) in isocratic mode using 0.1 mol/L NaCl solution. The flow rate of the mobile phase was 0.8 mL/min, and the column used was a TSK‐gel G2500PWXL (7.8 mm internal diameter (i.d.) × 30 cm; Tosoh Co., Ltd, Tokyo, Japan), which is generally used to fractionate substances with molecular weights from 100 to 5000. The elute was analyzed for nitrogen compounds using a flow‐through UV detector in scanning mode (190 nm to 400 nm) (Waters2996; Waters Co., Ltd).

The rest of the incubated meat was used in the analysis of the concentrations of IMP and Hx. The concentrations of IMP and Hx in the incubated meat were determined by the method of Kitada *et al.* (1983). The incubated meat (10 g) was homogenized in 25 mL of cold 10% perchloric acid using a homogenizer and then centrifuged at 5000 × *g,* 4°C for 10 min. The residue was mixed further with 20 mL of cold 10% perchloric acid and centrifuged again at 5000 × *g* for 5 min. Both supernatants were combined and the pH of the supernatants was adjusted to 6.4 using 5 N KOH and 100 mL distilled water. The solution was filtered using a 0.45 µm cellulose membrane and applied to an HPLC system in isocratic mode using the solvent consisting of 1% triethylamine‐phosphoric acid and 0.01% acetonitrile (pH 6.8). A Shimpack HRC‐ODS column was used (6.0 mm i.d. × 150 mm; Shimadzu, Kyoto, Japan). IMP and Hx were detected by a UV detector at 250 nm.

All chemical analyses were performed and repeated four times.

### Statistical analysis

In the sensory and chemical analyses, all data were expressed as the mean ± standard errors. The statistical significance of difference among means was evaluated using one‐sample *t*‐test at the 5% level.

## Results and Discussion

### Effect of HSSF on the enhancement of cured meat taste

**Figure 1:**
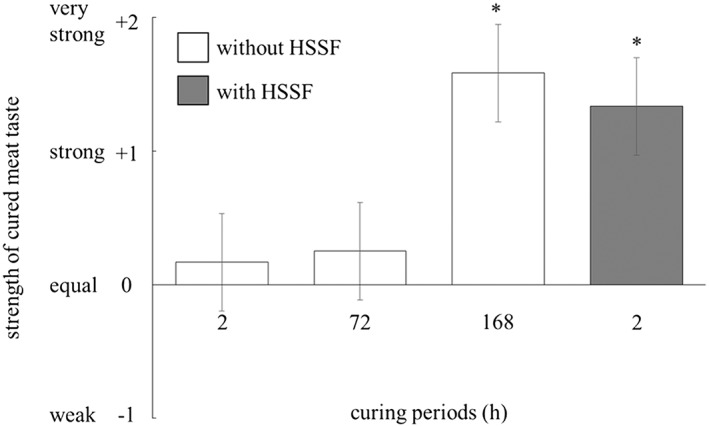
Effect of heat‐stable sarcoplasmic fraction (HSSF) on the strength of cured meat taste. The sliced meats were cured with the pickles for 2, 72 or 168 h or ones containing 50% (v/v) of HSSF for 2 h at 4°C, and then heated at 70°C for 20 min. The strength of the cured meat taste of the each cured sample was compared with that of the standard sample by sensory analysis. The values with asterisks are significantly different (*P* < 0.05). Histograms show mean values ± standard errors.

The sensory analysis for the sample cured with or without HSSF was performed. As the dry matter of HSSF contained more than 30% nitrogen (data not shown), nitrogen compounds seemed to be the important components of HSSF. To determine the effect of HSSF on the enhancement of cured meat taste, curing periods were set to 2 h in the samples cured with HSSF and to 2, 72 and 168 h in ones cured without HSSF samples (Fig. 1).

Samples cured with HSSF for 2 h exhibited a stronger taste profile than ones cured without HSSF for 72 h (*P* < 0.05). In contrast, the samples cured without HSSF for 168 h exhibited the same taste profile as ones cured with HSSF for 2 h. The long‐period incubation was necessary for the enhancement of the taste in the samples without HSSF. This result shows that some sarcoplasmic substances increase in raw meat during maturation, which were heat‐stable, water soluble and nitrogen‐containing compounds involved in the enhancement of taste. Nitrogen compounds contribute to meat taste (Yamasaki & Maekawa 1978; Nishimura & Kato 1988; Buscailhon *et al*. 1994; Kawai *et al*. 2002). Nishimura *et al*. (1988) investigated the changes in the levels of non‐protein compounds during post mortem aging, some of which are well known as being involved in the taste of food. In this investigation, the contents of free amino acids, small peptides, INO and Hx increased, while those of adenosine monophosphate and IMP decreased during post mortem aging. This finding suggested that the strengthening of the cured meat taste during maturation was induced by unknown nitrogen compounds contained in HSSF.

Moreover, the samples incubated with only 2% NaCl solution without nitrite did not exhibit a cured meat taste, regardless of the presence of HSSF (data not shown). Thus, nitrite was essential for the development of the cured meat taste. However, because the stronger taste was recognized in the samples cured with HSSF for only 2 h, nitrite itself might have little or no contribution to the enhancement of the taste during maturation.

### Identification of the compounds involved in the enhancement of cured meat taste

Because it is likely that nitrogen compounds contained in HSSF were involved in the enhancement of the cured meat taste in the preceding section, we examined the identification of those compounds using HSSF.

**Figure 2:**
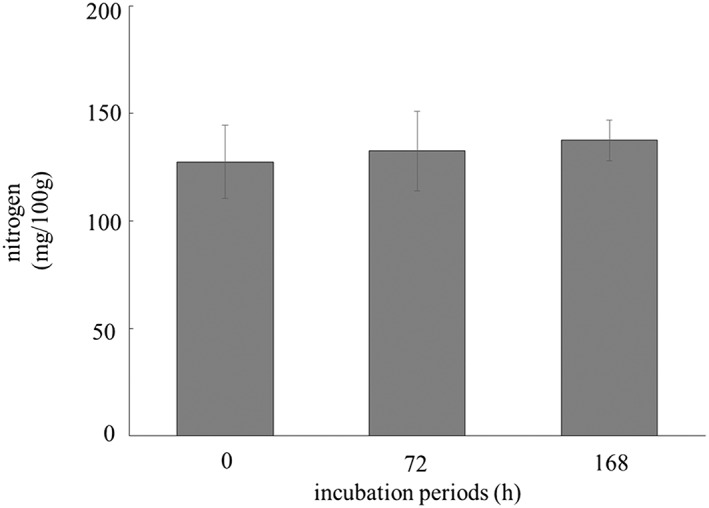
Changes on the concentration of nitrogen in heat‐stable sarcoplasmic fraction (HSSF) for various incubation periods. Minced meat incubated with an equal weight of 2% NaCl solution at 10°C for 0, 72 or 168 h were homogenized and centrifuged. The obtained supernatant was heated at 100°C for 20 min, and then centrifuged at 3000 × *g* for 10 min. The concentration of nitrogen in the resultant supernatant (HSSF) was determined by the combustion method. Histograms show mean values ± standard errors.

First, we focused on the changes in the concentration of nitrogen compounds in HSSF prepared from the meat incubated with 2% NaCl solution. Figure 2 shows the nitrogen concentrations in HSSF at various incubation periods.

The nitrogen concentrations in HSSF did not change during the incubation from 0 to 168 h. These results imply that macro‐molecular compounds such as proteins in meat were not degraded to generate soluble low molecular weight compounds, such as amino acids or small peptides, during the incubation.

Second, the behavior of nitrogen compounds in HSSF during the incubation from 0 to 168 h was examined by gel filtration chromatography. There were no changes in the chromatograms of the fractions monitored at 220 or 280 nm, related to amino acids and small peptides (data not shown). This implies that the insoluble structural proteins such as myofibrillar or connective tissue proteins or free soluble proteins were not degraded to soluble amino acids or small peptides.

**Figure 3:**
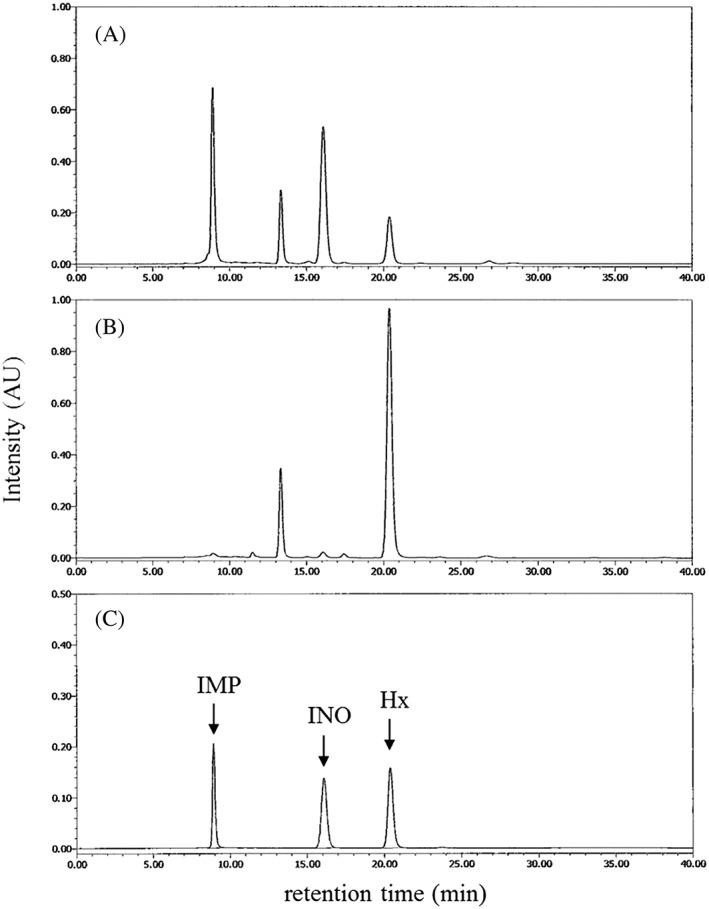
Gel filtration chromatograms of the fractions of heat‐stable sarcoplasmic fraction (HSSF) monitored at 250 nm. A fractionation of nitrogen compounds in HSSF prepared from minced meat incubated for 0 (A) or 168 (B) h at 10°C was carried out by gel filtration chromatography. The changes of nitrogen compounds were detected by a spectrophotometer using 250 nm. The mixture of calibration substances of typical adenosine triphosphate (ATP)‐related compounds included in meat, inosine monophosphate (IMP), inosine (INO) and hypoxanthine (Hx), was also applied to gel filtration chromatography in (C).

On the other hand, the chromatograms of the fractions monitored at 250 nm, which are related in ATP‐related compounds, visibly changed during incubation from 0 to 168 h as shown in Figure 3. The comparison of chromatograms of HSSF incubated for 0 (Fig. 3A) and 168 h (Fig. 3B) showed that the peaks of first and third fractions of HSSF decreased during incubation and almost completely disappeared.

In contrast, the peak area of the forth fraction increased in HSSF incubated for 168 h (Fig. 3B). To identify the main compounds constituting the forth fraction, the chromatogram of the forth fraction was compared with that of the ATP‐related compounds, IMP, INO and Hx, which are included in meat (Fig. 3C). The retention time of the fourth fraction corresponded to that of Hx. The peak of the first fraction that disappeared by 168 h of incubation corresponded to that of IMP (Fig. 3C). IMP is known to be a major component of taste in foods. Hx is produced by the degradation of IMP during post mortem aging of meat, and is used as an indicator of putrefaction in fish meat (Hughes & Jones 1966). The relationship between IMP and Hx in the development of the cured meat taste remains unresolved.

**Figure 4:**
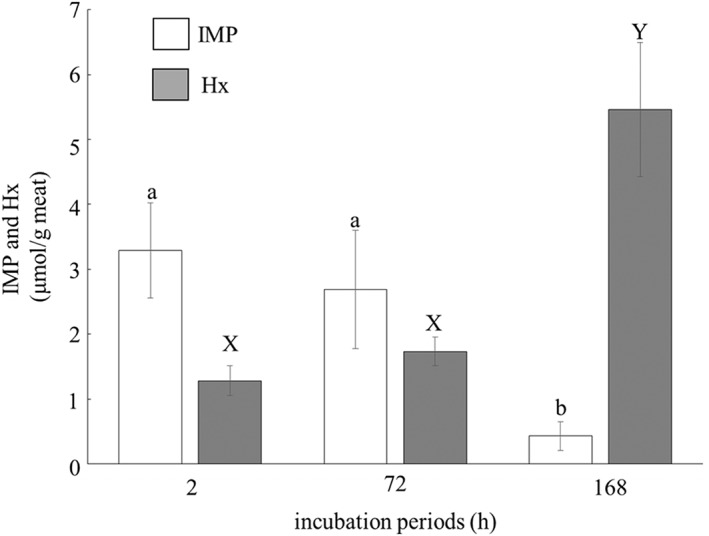
Changes on the concentration of inosine monophosphate (IMP) and hypoxanthine (Hx) in the meat incubated with the 2% NaCl solution for various periods. Minced meat was mixed to an equal weight of 2% NaCl solution, and then incubated at 10°C for 0, 72 or 168 h. The concentrations of IMP and Hx in the incubated meat were analyzed by the method of Kitada *et al*. (1983). Histograms show mean values ± standard errors. Bars with different capital and lowercase letters are significantly different (*P* < 0.05).

Third, the changes in the concentrations of Hx and IMP in the minced meat during incubation with 2% NaCl solution for 0, 72 and 168 h are presented in Figure 4.

The concentration of Hx gradually increased and that of IMP decreased during incubation from 0 to 168 h. The concentration of Hx in the incubated meat was lower than that of IMP at incubation for 72 h, but increased and reached 5.5 µmol/g meat at 168 h incubation (*P* < 0.05). This implies that the marked increase in the concentration of Hx was induced by the degradation of IMP during incubation with 2% NaCl solution. So far, Hx has not been demonstrated to contribute to the taste of meat. Nishimura (1988) insisted that INO and Hx did not contribute to the improvement of taste during storage, on the basis of the report that these compounds have no effect on taste (Hayashi *et al*. 1981). On the other hand, IMP is an ‘umami’ compound of foods, as well as glutamic acid (Yamaguchi 1991; Kurihara & Kashiwayanagi 2000; Yamaguchi & Ninomiya 2000). It was reported that glutamic acid, IMP and potassium ion contribute to the taste of chicken meat (Fujimura *et al*. 1995). However, there is a report that the concentration of IMP decreases during curing maturation (Escudero *et al*. 2011). In the present study also, IMP concentration decreased to < 1.0 µmol/g meat at 168 h incubation. These data suggest that the contribution of Hx was predominant compared to IMP on the enhancement of cured meat taste. This is consistent with a previous study that the concentration of IMP was low and that of Hx was high in meat products cured for long periods (Escudero *et al*. 2011).

### Effect of Hx on enhancement of cured meat taste

**Figure 5:**
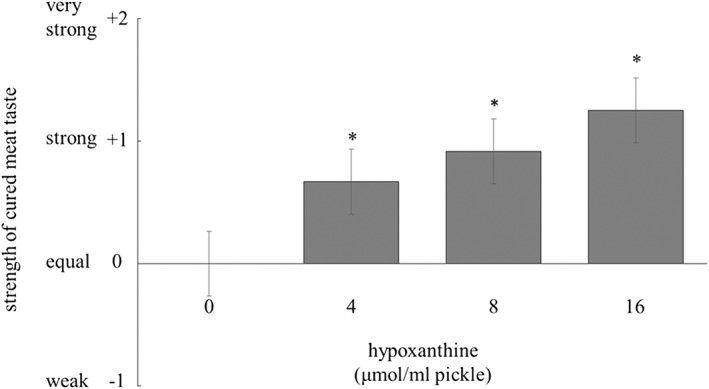
Effect of hypoxanthine (Hx) on the strength of cured meat taste. The sliced meat was cured with the pickle consisting of 2% NaCl, 200 ppm NaNO_2_ and various concentrations of Hx (0, 4, 8 and 16 µmol/mL) at 4°C for 2 h, and then heated at 70 °C for 20 min. The strength of cured meat taste of the samples with Hx was compared with that of the standard ones without Hx by sensory analysis. The values with asterisks are significantly different (*P* < 0.05). Histograms show mean values ± standard errors.

To clarify the contribution of Hx in the enhancement of cured meat taste during maturation, the samples cured with the pickles containing various concentration of Hx were subjected to sensory analysis. Figure 5 shows the effect of Hx on the enhancement of the cured meat taste.

The addition of Hx, in a dose‐dependent fashion, enhanced the cured meat taste by maturation for 2 h. When the concentration of Hx was higher than 4 µmol/mL in the pickle, there were significant differences in the strength of cured meat taste compared with the standard sample (*P* < 0.05). The differences tended to be dependent on the concentration of Hx added to the pickle, albeit not at significant levels. Table 1 shows the concentrations of Hx in the samples cured with the pickle containing Hx. The concentration of Hx increased dose‐dependently by the addition of Hx, while that of IMP was constant.

**Table 1:** Changes in contents of hypoxanthine (Hx) in samples during the addition of Hx into pickle

Hx contents					
Pickle†	(µmol/mL)	0	4	8	16
Sample‡	(µmol/g)	0.72	2.40	3.70	7.03

Values are expressed as mean of two samples.

† Concentration of Hx added to pickle

‡ Concentration of Hx in samples cured with the pickle

Because the concentration of Hx in the meat incubated with 2% NaCl solution for 2 h was less than 2.0 µmol/mg meat (Fig. 4), that of Hx in HSSF was also assumed to be at a low level. The concentration of Hx in samples cured with the pickle without Hx for 2 h was 0.72 µmol/mg meat (Table 1). Nevertheless, samples cured with the pickles containing HSSF for 2 h exhibited a stronger taste profile (Fig. 1). This suggests that there were components other than Hx involved in the stronger taste. Many nitrogen compounds and various minerals related to taste of food may be contained in HSSF. Also, there is a report that potassium ion contributes to the taste of chicken meat (Fujimura *et al*. 1995). Therefore, it seems that the prominent effect of the low concentration of Hx in HSSF was induced by synergy between Hx and other components related to cured meat taste in HSSF, and the synergy lowered the threshold for the effect of Hx alone.

From the present study, it was concluded that Hx is essential for the enhancement of cured meat taste during maturation. Hx has not been studied as a substance participating in the taste of meats. Because Hx is a bitter substance (Tikk *et al*. 2006), the bitter taste inherent to Hx may contribute to the high palatability in cured meat.

To clarify of the mechanism of the enhancement of cured meat taste induced by Hx, we have to elucidate the possibilities of the production of new taste compounds brought about by the reaction of nitrite with Hx, and the participation of factors other than nitrite and Hx, the interactions and synergies between these factors, and the effect of a bitter taste inherent to Hx on the taste of cured meat products.
